# Sexual prejudice and associated factors among clinical nurses in Türkiye: A cross-sectional study

**DOI:** 10.1371/journal.pone.0360301

**Published:** 2026-10-08

**Authors:** Ezgi Şişman, Mehtap Güngör, Umut Günay, Aila Gareayaghi, Hanife Yılmaz, İlay Dalkıran, Fatma Seher Kocaayan, Aslıhan Polat

**Affiliations:** 1 Department of Psychiatry, Faculty of Medicine, Kocaeli University, İzmit, Kocaeli, Türkiye; 2 Psychiatry Clinic, Kocaeli City Hospital, İzmit, Kocaeli, Türkiye; 3 Şemdinli State Hospital, Şemdinli, Hakkari, Türkiye; 4 Community Mental Health Center, Kocaeli University, İzmit, Kocaeli, Türkiye; University of Technology Sydney, AUSTRALIA

## Abstract

**Aim/objective:**

This study aimed to assess the level of homophobia and to identify its independent predictors among practicing clinical nurses working in a tertiary university hospital in Türkiye.

**Background:**

Homophobia among healthcare professionals undermines equitable care for sexual minorities and remains underexplored in Türkiye, particularly among practicing nurses. In settings with limited institutional support for sexual minority health, identifying determinants of sexual prejudice among healthcare professionals can inform training needs.

**Design:**

A cross-sectional survey design was used.

**Methods:**

One hundred registered nurses providing direct patient care completed the Turkish version of the Hudson and Ricketts Homophobia Scale and a structured questionnaire assessing sociodemographic characteristics, knowledge, etiological beliefs, interpersonal contact, and comfort working with LGB patients and colleagues. Homophobia scores were dichotomized using a median split, and binary logistic regression was performed to identify independent predictors

**Results:**

The mean homophobia score was 102.14 (SD = 24.73). In multivariate analysis, endorsing biological explanations for homosexuality (OR = 0.22), having a homosexual acquaintance (OR = 0.19) and greater comfort working with LGB colleagues (OR = 0.41) were independently associated with lower odds of homophobia, whereas holding a bachelor’s degree was associated with higher odds (OR = 4.24).

**Conclusions:**

Homophobia scores among nurses were shaped by educational, interpersonal, cognitive, and affective factors. These findings underscore the need for structured, evidence-based, and inclusive nursing education and institutional reform to promote equitable and ethical care for sexual minority patients.

## Introduction

Recent global estimates suggest that approximately 9% of adults identify as members of the LGBTQ+ (lesbian, gay, bisexual, transgender, queer, and additional sexual and gender minority populations) community. While the visibility of LGBTQ+ individuals remains relatively limited in certain countries—such as Japan, South Korea, Türkiye, Romania, Hungary, and Poland—survey data indicate that around 6% of adults in Türkiye self-identify as lesbian, gay, or bisexual [1]. Parallel to the growing number of individuals identifying as LGBTQ + , a gradual increase in the societal acceptance of homosexuality has also been documented in recent years [2].

Although significant legal and normative advances have been made globally regarding the rights of LGBTQ+ individuals, public attitudes toward homosexuality continue to vary widely across countries, shaped by geographic, economic, political, and religious factors. In recent years, Türkiye has experienced a notable shift toward more conservative sociopolitical orientations, with legislative discourse increasingly reflecting traditionalist Islamic values. As a result, Türkiye received a score of only 5 out of 100 in ILGA-Europe’s 2026 Rainbow Map, ranking among the three lowest countries in Europe alongside Russia and Azerbaijan, performing poorly across all indicators related to LGBTQ+ rights [3]. Consistent with broader international patterns of divergence in societal acceptance, only 25% of respondents in Türkiye agreed that homosexuality should be accepted by society [2]. Furthermore, unlike the upward trends observed in support for same-sex marriage in many countries, Türkiye showed a modest decline from 24% in 2021 to 20% in 2023 [1].

Despite limited public visibility, the LGBTQ+ community in Türkiye has increasingly become the focus of negative political discourse and institutional rhetoric in recent years. Since 2015, Pride parades have been officially banned, while state-sanctioned rallies opposing LGBTQ+ rights have gained visibility. Senior government officials and official national campaigns have increasingly framed LGBTQ+ identities as a threat to the traditional family structure [4, 5].

Türkiye may be characterized as a society marked by competing forces of traditionalism and modernity, with tensions between these orientations becoming increasingly pronounced over the past decade. Within this sociopolitical landscape, the LGBTQ+ population appears to occupy a position at the outer margins of a continuum defined by a conservative power base and a secular minority. Historically, nonconformity to dominant norms of masculinity has often been met with social repression, a pattern that has become more visible within political discourse in recent years. Although homosexuality is not criminalized under Turkish law, and the Constitution of the Republic of Türkiye guarantees equality before the law, the absence of explicit protections related to sexual orientation limits the practical applicability of these constitutional rights for LGBTQ+ individuals [6]. In contrast to advancements made at the European Union and United Nations levels, Türkiye lacks comprehensive anti-discrimination legislation addressing sexual orientation and gender identity [7]. Recent legislative proposals have sought to further restrict the public expression of LGBTQ+ rights [8].

It is well-established that in societies where heterosexuality is constructed as the norm, LGBTQ+ individuals are routinely subjected to stereotypical perceptions and face multifaceted inequalities rooted in stigma and discrimination—spanning social exclusion, political marginalization, and healthcare disparities [9,10]. In addition to their specific health needs, LGBTQ+ populations experience significantly greater health disparities compared to cisgender, heterosexual individuals, including elevated rates of chronic physical conditions such as pain, cardiovascular disease, cognitive decline, and various cancers [11]. These disparities do not stem from sexual orientation itself, but from the stigma and discrimination that sexual and gender minorities encounter across social institutions [12]. According to the minority stress framework, sustained exposure to this stigma generates cumulative psychosocial stress that adversely affects health [13]. Such stigma does not stop at the doors of healthcare institutions; providers are themselves members of the society in which they live and may carry its prevailing prejudices, often unknowingly, into their encounters with patients [14]. Discrimination experienced in the wider society is thereby reproduced within care settings, which is precisely what makes healthcare professionals’ attitudes a decisive part of the disparities in question. At the same time, contemporary LGBTQ+ health care faces a global crisis, with the majority of providers lacking adequate knowledge of LGBTQ+ health issues [15,16]. This gap is largely attributable to the persistent exclusion of sexual and gender minority content from traditional medical and nursing curricula [17].

Frequently overlooked in clinical settings, LGBTQ+ individuals are widely recognized as a priority population in discussions of healthcare disparities. The social inequities affecting this group—particularly those related to the distribution of power, access, and opportunity—are not incidental but reflect structural injustices that have been systematically reinforced over time. Within this context, growing sociopolitical conservatism, both in Türkiye and across parts of the Western world, has raised concerns about the normalization of discriminatory attitudes toward LGBTQ+ individuals, even within healthcare environments. The U.S. Department of Health and Human Services has formally identified the LGBTQ+ population as vulnerable and called for systematic data collection regarding patients’ sexual orientation and gender identity [18]. As frontline providers, nurses often constitute the first point of contact in the healthcare system and thus play a critical role in shaping patient experiences [19]. Professional guidelines from the American Nurses Association [20], the Joint Commission [21], and the Turkish Nurses Association [22] explicitly mandate the delivery of culturally competent and equitable care to all individuals, including those from sexual and gender minority groups. However, nurses’ attitudes frequently reflect broader societal ideologies—including racism, sexism, and homophobia—which may inadvertently influence clinical interactions [23]. Homophobia refers here to negative attitudes and affective reactions toward homosexual individuals, as assessed in the present study [24], whereas heteronormativity describes the broader social and institutional assumption that heterosexuality constitutes the default and expected form of sexuality [25]. Consequently, prevailing homophobia and heteronormativity in the larger sociocultural context may also be mirrored within the nursing profession itself [26–28].

Nursing has a well-established tradition of engagement with social critique, advocacy, and policy reform—an orientation broadly conceptualized as social ethics [29]. Nevertheless, nurses, like other healthcare professionals, are not exempt from the intersecting systems of oppression embedded in the societies in which they are trained and practice. These structural influences can shape internalized biases that may, consciously or unconsciously, translate into discriminatory behaviors in clinical care [30,31].

Research addressing LGBTQ+ health within the context of nursing education and practice remains limited and is predominantly concentrated in Western countries [32]. Globally, studies examining homophobia among nurses are relatively scarce, and such research is even more limited within the Turkish context. This paucity of scholarship in Türkiye is shaped by a range of factors, including a highly polarized sociopolitical environment that presents both structural and cultural barriers to conducting research in this area. The absence of institutional support for LGBTQ+ health research, coupled with challenges in securing administrative approval and reluctance among healthcare professionals to participate—often due to prevailing prejudices or concerns regarding professional repercussions—further complicates empirical inquiry [33–35].

Among all healthcare professionals, nurses typically spend the most time in direct patient interaction. This positions them uniquely as potential agents of change in promoting equitable care for LGBTQ+ individuals—provided they are equipped with both evidence-based knowledge and critical self-awareness. Given their central role in patient care, it is essential that nurses are capable of delivering competent and inclusive services to all patients, regardless of sexual orientation or gender identity. In Türkiye, dedicated institutional structures addressing LGBTQ+ health remain scarce. Notably, Kocaeli University Hospital houses one of the country’s few Multidisciplinary Gender Clinics, where nurses frequently conduct initial health assessments—an important opportunity to gather relevant demographic and psychosocial data. Competency in understanding and addressing the needs of diverse populations is vital in these settings [36]. A lack of familiarity with LGBTQ-related terminology or issues may contribute to suboptimal care, reliance on stereotypes, and the inadvertent marginalization of patients [37,38]. While recognizing terminology is a fundamental first step, structural changes within healthcare institutions are also necessary to remove systemic barriers to equitable care. Alongside such structural measures, current evidence indicates that educational and contact-based interventions can improve healthcare providers’ knowledge, attitudes, and comfort in working with sexual and gender minority patients [39,40].

In light of these challenges, we designed a structured training program for practicing nurses at our university hospital to enhance their competence in LGBTQ + -related health issues and to mitigate the effects of minority stress [13] that LGBTQ+ patients may experience during healthcare encounters. Given the limited body of research on homophobia among nurses in Türkiye, the present study aimed to assess the level of homophobia and its predictors among hospital-based nurses (including those involved in the supervision of nursing interns), as well as their knowledge, attitudes, self-reported competence, willingness, and behaviors related to caring for LGBTQ+ individuals prior to attending the training.

## Materials and methods

### Study design and setting

This cross-sectional study was conducted between December 1, 2022 and February 1, 2023 at a tertiary university hospital in Türkiye, located in a densely populated, highly industrialized province in the eastern Marmara region of northwestern Türkiye, within the greater Istanbul industrial corridor. This research was conducted as part of a broader institutional effort to evaluate and enhance healthcare providers’ preparedness for delivering inclusive care to sexual and gender minority populations. The study was coordinated by the Department of Psychiatry and implemented in collaboration with the hospital’s Nursing Directorate, which supported participant recruitment and facilitated data collection.

The present survey served as the baseline assessment of this institutional in-service training initiative. The training was delivered at the hospital as a single, 3-hour, in-person session by psychiatrists and psychiatric nurses affiliated with the university’s Department of Psychiatry and its Gender Dysphoria Clinic, using a standardised slide set. The curriculum covered core terminology and conceptual distinctions (gender identity versus sexual orientation; gender identity versus biological sex), gender dysphoria, sexual orientation and its aetiology, principles of respectful clinical communication, and the specific healthcare needs of transgender patients. All data reported in the present study were collected before participants were exposed to any training content.

### Participants and sampling strategy

The study sample consisted of 100 registered nurses who were actively providing direct patient care across various clinical departments, including internal medicine, surgery, emergency, and psychiatry. Participants were randomly selected by the hospital’s Nursing Directorate from eligible nursing staff across the four clinical departments (internal medicine, surgery, emergency, and psychiatry) to ensure representation across departments; the investigators played no role in identifying or approaching individual nurses. Inclusion criteria included (1) being a currently employed nurse at the institution, (2) providing direct clinical care, and (3) not having participated in similar prior surveys. Incomplete questionnaires were to be excluded from the analysis; however, all 100 returned questionnaires were fully completed, and no participant was excluded on this basis. There were no missing data.

Participants received sealed paper questionnaires that they completed privately at home and returned anonymously to a locked drop-box within the nursing directorate. The anonymity of responses was strictly maintained. All 100 distributed questionnaires were returned fully completed, yielding a 100% response rate. The dataset used in the present study is part of a larger cross-sectional project investigating attitudes toward sexual and gender minorities among healthcare professionals. Findings focusing specifically on transphobia within the same sample have been published previously [41].

### Ethical considerations

Ethical approval was obtained from the Kocaeli University Non-Interventional Clinical Research Ethics Committee (approval no: KÜ GOKAEK 01.4; decision no. 8.03; project no. 2019/154; approved 16 May 2019). All procedures were conducted in accordance with the Declaration of Helsinki. Written informed consent was obtained from all participants. Participation was entirely voluntary, and participants were informed that their responses would remain confidential and would not influence their employment status.

### Measures

#### Sociodemographic and background variables.

A structured information form was used to collect sociodemographic and experiential data. Variables included age, gender, marital status, level of education, years of professional experience, clinical unit, and religious orientation. Religious orientation was assessed using a single self-identification item with three response options: non-believer, believer, and religiously conservative. The last category reflected participants who self-identified as “conservative” rather than simply as “believer”; it was not derived from a standardized measure of religiosity. Participants were also asked whether they had previously worked in psychiatric settings, knew someone who identified as LGB (lesbian, gay, and bisexual), or had received any education on LGB-related health issues. Participants were also asked whether they believed homosexuality to be biological in origin — that is, innate rather than acquired — or environmental/temporary. This item reflects participants’ beliefs and is not a statement about the aetiology of sexual orientation. Additionally, family-level attitudes were assessed via a single-item measure of how topics related to sexual orientation were approached in the participant’s household (negative, neutral, or supportive).

#### Perceived and objective knowledge, and comfort in clinical settings.

Participants were asked to rate their subjective comfort levels when working with LGB patients and colleagues using a 5-point Likert scale (1 = very uncomfortable to 5 = very comfortable). These items aimed to capture emotional readiness and interpersonal openness in clinical contexts. Self-perceived knowledge of LGB health was also assessed on a 5-point scale, reflecting subjective confidence rather than formal training. Objective knowledge was measured using a set of six multiple-choice questions to assess conceptual understanding of sexual orientation and related terminology (e.g., homosexuality, bisexuality, heterosexuality).

#### Homophobia.

Homophobia was assessed using the Hudson and Ricketts Homophobia Scale, a 25-item instrument originally developed to measure negative attitudes toward homosexual individuals [24]. In this study, the 24-item Turkish version adapted by Sakallı and Uğurlu was utilized [42]. This version has demonstrated excellent internal consistency in prior research, with a Cronbach’s alpha coefficient of 0.94. In the current study, only the total score was used.

Participants were asked to rate each item on a 6-point Likert scale ranging from 1 (strongly disagree) to 6 (strongly agree). Higher scores on the scale indicate greater levels of homophobia. The total homophobia score was used in subsequent analyses as the primary outcome variable. Total scores on the Turkish version of the scale can range from 24 to 144, with higher scores indicating greater homophobia. A median split was applied to the total score to categorise participants into a lower HRHS score group (<104) and a higher HRHS score group (≥104) relative to the sample median. The scale has no validated cutoff distinguishing “low” from “high” homophobia; these labels therefore denote relative position within the present sample only. While the Hudson and Ricketts Homophobia Scale does not provide established cutoff scores, previous studies have adopted a similar median-based grouping strategy for comparative purposes, especially in the absence of normative thresholds or when the score distribution deviates from normality [43].

### Statistical analysis

All statistical analyses were conducted using IBM SPSS Statistics for Windows, Version 22.0. A total of 100 participants were included in the analysis, with no missing data for the primary outcome variable or key predictors. Descriptive statistics were used to summarize the sociodemographic characteristics and primary study variables. Continuous variables are summarised as means and standard deviations. Frequencies and percentages were calculated for categorical variables.

The primary outcome measure—the total score on the Hudson and Ricketts Homophobia Scale—was assessed for normality using both the Shapiro–Wilk test and the D’Agostino–Pearson omnibus test. Results indicated significant deviation from a normal distribution (Shapiro–Wilk p = 0.012; D’Agostino–Pearson p = 0.047), with a slight left skew (skewness = –0.39). Based on these findings, the total homophobia scores were dichotomized at the median value (104 points) to create a binary classification suitable for logistic regression modeling. This approach was specified a priori and reflects the applied aim of the study: as a baseline assessment preceding a structured training programme, the primary question was which nurses were more likely to fall within the higher-prejudice group and would therefore benefit most from targeted intervention, rather than the number of scale points separating the groups. The scale also has no established normative cut-off.

To explore bivariate associations between homophobia classification and categorical independent variables (e.g., marital status, religious beliefs, acquaintance with homosexual individuals), Pearson’s chi-square or Fisher’s exact tests were used, as appropriate. For continuous variables such as age and years of professional experience, the Mann–Whitney U test was applied due to the non-normal distribution of the data.

Independent predictors of homophobia were subsequently examined using binary logistic regression analysis. All variables with significant group-level differences and/or established empirical relevance based on prior literature (e.g., education, interpersonal contact, etiological beliefs) were entered into the logistic regression model to examine their independent associations with homophobia. Odds ratios (ORs) with 95% confidence intervals (CIs) were calculated to estimate the strength and direction of associations. Model performance was evaluated using Nagelkerke R² and classification accuracy. To further assess model fit, the Hosmer–Lemeshow goodness-of-fit test was conducted. A confusion matrix was also generated to examine sensitivity and specificity. Results from these analyses are presented in the Results section. Prior to model construction, multicollinearity was evaluated using variance inflation factor (VIF) diagnostics; all VIF values were below 3.0, with most under 1.2, indicating no collinearity concerns among predictors.

A post hoc power analysis using G*Power 3.1 indicated that the sample size of 100 provided sufficient power (1–β = 0.94) to detect medium effect sizes (OR ≈ 2.5) in a binary logistic regression model with seven predictors, assuming α = 0.05. This finding confirms that the sample size was adequate to detect meaningful associations in the regression analysis.

## Results

### Descriptive characteristics of the sample

The study sample consisted of 100 nurses, of whom 93.0% were female and 7.0% were male. The mean age was 29.86 years (SD = 5.98), and the average professional experience was 8.04 years (SD = 5.76). Most participants were married (59.0%) and held a bachelor’s degree (63.0%). Only 7.0% reported having previously worked in a psychiatric unit.

In terms of birthplace, 65.0% of participants were born in urban areas and 35.0% in rural areas. Regarding sexual orientation, 99.0% identified as heterosexual and 1.0% as homosexual. When asked about religious orientation, 90.0% identified as believers, 5.0% as non-believers, and 5.0% as religiously conservative. A total of 21.0% of participants indicated having a homosexual acquaintance. Regarding family dynamics, 46.0% stated that sexual topics were never discussed in the household, while 37.0% reported that they were rarely discussed and 17.0% reported occasional discussions. When asked about parental attitudes toward LGB individuals, 63.0% of participants reported negative views and 37.0% reported neutral attitudes; none indicated positive parental attitudes.

Beliefs about the etiology of homosexuality varied, with 60.0% attributing it to biological causes and 40.0% to environmental or temporary factors. Self-perceived knowledge on homosexual health issues was limited: 25.0% reported having no knowledge, 45.0% a little, and 30.0% moderate knowledge; no participants reported good or expert-level knowledge.

Regarding comfort levels, 37.0% agreed or strongly agreed with the statement “I feel comfortable working with homosexual patients,” while 41.0% were neutral and 22.0% expressed discomfort. Similarly, when asked about working with LGB colleagues, 33.0% expressed agreement, 33.0% were neutral, and 34.0% expressed discomfort.

The mean score on the Hudson and Ricketts Homophobia Scale was 102.14 (SD = 24.73), with scores ranging from 43.00 to 141.00. Sociodemographic characteristics are summarized in Table 1.

**Table 1:** Sociodemographic, educational, and professional characteristics of the nursing sample.

Categorical Variable	Response	n (%)
Gender	Female	93 (93.0%)
	Male	7 (7.0%)
Marital status	Married	59 (59.0%)
	Single	41 (41.0%)
Education level	High school/ Associate degree	25 (25.0%)
	Bachelor’s degree	63 (63.0%)
	Master’s or higher	12 (12.0%)
Experience working in psychiatry unit	Yes	7 (7.0%)
General religious identification	Non-believer	5 (5.0%)
	Believer	90 (90.0%)
	Religiously conservative	5 (5.0%)
Any homosexual acquaintance	Yes	21 (21.0%)
Parental attitudes toward LGB individuals	Negative	63 (63.0%)
	Neutral	37 (37.0%)
Discussion of sexual topics within the family	Never	46 (46.0%)
	Rarely	37 (37.0%)
	Occasionally	17 (17.0%)
Beliefs about etiology of homosexuality	Biological	60 (60.0%)
	Environmental/Temporary	40 (40.0%)
Concept: Heterosexuality	Correct response	87 (87.0%)
Concept: Homosexuality	Correct response	86 (86.0%)
Concept: Bisexuality	Correct response	73 (73.0%)
Perceived knowledge on homosexuality	None	25 (25.0%)
	A little	45 (45.0%)
	Moderate	30 (30.0%)
Willingness to receive LGB-related training	Yes	64 (64.0%)
Comfort working with homosexual patients	Yes	37 (37.0%)
Comfort working with LGB colleagues	Yes	33 (33.0%)
Numerical Variable	**Mean ± SD**	**Range**
Age	29.86 ± 5.98	20.00–46.00
Years of professional experience	8.04 ± 5.76	0.50–25.00
Hudson and Ricketts homophobia scale score	102.14 ± 24.73	43.00–141.00

### Group comparisons based on median-split HRHS score

Group-based comparisons are presented in Table 2. Marital status significantly differed between groups, with a higher proportion of married participants in the higher HRHS score group(p = .015). A statistically significant association was also observed for having a homosexual acquaintance (p = .003), with the lower HRHS score groupmore frequently reporting such contact. Parental attitudes toward LGB individuals also differed significantly between groups, with negative attitudes more common in the higher HRHS score group (74.0% vs. 52.0%; p = .038). Beliefs regarding the etiology of homosexuality differed significantly across groups; those in the lower HRHS score groupmore commonly endorsed biological explanations (p = .025).

**Table 2:** Group Comparisons Based on Homophobia Classification.

Variable	Subgroup	Lower HRHS scoreN(%) (<104)	Higher HRHS scoreN(%) (≥104)	p value
Gender	Female	46(92.0%)	47(94.0%)	1.000
	Male	4(8.0%)	3(6.0%)	
Marital status	Single	27(54.0%)	14(28.0%)	**0.015**
	Married	23(46.0%)	36(72.0%)	
Education level	High school&Associate deg.	14(28.0%)	11(22.0%)	0.291
	Bachelor’s degree	28(56.0%)	35(70.0%)	
	Master’s or higher	8(16.0%)	4(8.0%)	
Experience working in psychiatry unit	Yes	3(6.0%)	4(8.0%)	1.000
	No	47(94.0%)	46(92.0%)	
Religious belief	Religiously conservative	1(2.0%)	4(8.0%)	0.360
	Non-believer	3(6.0%)	2(4.0%)	
	Believer	46(92.0%)	44(88.0%)	
Any homosexual acquaintance	Yes	17(34.0%)	4(8.0%)	**0.003**
	No	33(66.0%)	46(92.0%)	
Discussion of sexual topics within the family	Never	22(44.0%)	24(48.0%)	0.725
	Rarely	18(36.0%)	19(38.0%)	
	Occasionally	10(20.0%)	7(14.0%)	
Parental attitudes toward LGB individuals	Negative	26(52.0%)	37(74.0%)	0.038
	Neutral	24(48.0%)	13(26.0%)	
Etiology of homosexuality	Biological	36(72.0%)	24(48.0%)	**0.025**
	Environmental	14 (28.0%)	26 (52.0%)	
Concept: Heterosexuality	Correct response	47(94.0%)	40(80.0%)	**0.074**
	Incorrect response	3(6.0%)	10(20.0%)	
Concept: Homosexuality	Correct response	44(88.0%)	42(84.0%)	0.773
	Incorrect response	6(12.0%)	8(16.0%)	
Concept: Bisexuality	Correct response	39(78.0%)	34(68.0%)	0.368
	Incorrect response	11(22.0%)	16(32.0%)	
Comfort working with homosexual patients	Disagree	4(8.0%)	18(36.0%)	**<.001**
	Neutral	18(36.0%)	23(46.0%)	
	Agree	28(56.0%)	9(18.0%)	
Comfort working with LGB colleagues	Disagree	10(20.0%)	24(48.0%)	**<.001**
	Neutral	12(24.0%)	21(42.0%)	
	Agree	28(56.0%)	5(10.0%)	

No significant group differences were observed for the concepts of heterosexuality, homosexuality, or bisexuality (all p > .05).Self-reported comfort working with LGB patients and LGB colleagues differed significantly by HRHS score group (p < .001 for both). Nurses in the lower HRHS score group more frequently reported agreement with comfort-related items, while those in the higher HRHS score groupmore commonly indicated discomfort or neutrality.

Group-level differences in variables such as gender, education level, experience in psychiatric units, religious identification and discussion of sexual topics within the family did not reach statistical significance (all p > .05).

### Predictors of homophobia: Binary logistic regression analysis

The results of the binary logistic regression model examining predictors of homophobia are presented in Table 3. Education level did not reach significance as an overall predictor (Wald = 5.190, df = 2, p = .075). Specifically, having a bachelor’s degree was associated with significantly increased odds of being in the higher HRHS score group compared to participants with only high school or associate degrees (B = 1.444, p = .036, OR = 4.238, 95% CI [1.099, 16.342]). No significant difference was found for those with a master’s degree or higher.

**Table 3:** Predictors of Homophobia: Binary Logistic Regression Analysis.

Independent Variables	B	Std. Error	Wald	p	OR (Exp(B))	95% CI for OR (Lower–Upper)
Education level: Bachelor’s degree (ref: high school/ associate)	1.444	0.689	4.397	**0.036**	4.238	1.099–16.342
Education level: Master’s or higher (ref: high school/ associate)	0.226	1.035	0.048	0.827	1.254	0.165–9.525
Any homosexual acquaintance (yes)	−1.683	0.786	4.583	**0.032**	0.186	0.040–0.868
Belief about the etiology of homosexuality (biological vs. environmental)	−1.512	0.607	6.203	**0.013**	0.220	0.067–0.725
Marital status (married vs. single)	1.087	0.573	3.594	0.058	2.964	0.964–9.118
Willingness to receive LGB-related training (yes)	−0.980	0.610	2.583	0.108	0.375	0.114–1.240
Comfort working with homosexual patients	−0.444	0.388	1.311	0.252	0.642	0.300–1.372
Comfort working with LGB colleagues	−0.887	0.377	5.528	**0.019**	0.412	0.197–0.863
Constant	4.217	1.251	11.361	<.001	67.842	–

Note: Odds ratios refer to the odds of membership in the higher HRHS score group (≥104). OR > 1 indicates greater odds of higher homophobia; OR < 1 indicates lower odds.

Participants who reported having any homosexual acquaintance had significantly lower odds of membership in the higher HRHS score group (B = −1.683, p = .032, OR = 0.186, 95% CI [0.040–0.868]). Participants who endorsed a biological explanation for the etiology of homosexuality had significantly lower odds of being in the higher HRHS score group (B = −1.512, p = .013, OR = 0.220, 95% CI [0.067–0.725]).

Comfort working with LGB colleagues also remained a significant predictor. Higher comfort levels were associated with lower odds of homophobia (B = −0.887, p = .019, OR = 0.412, 95% CI [0.197, 0.863]).

Other predictors, including marital status, willingness to receive LGB-related training, and comfort working with homosexual patients, were not statistically significant in the model. The model achieved an overall classification accuracy of 80.0%, correctly classifying 72.0% of participants with homophobia scores below the median and 88.0% of those with scores above the median. Model calibration was acceptable based on the nonsignificant Hosmer–Lemeshow goodness-of-fit test (χ² = 8.95, df = 8, p = 0.347). Full regression results are provided in Table 3.

## Discussion

This study identified key factors associated with elevated homophobia scores among nurses, based on responses to the Hudson and Ricketts Homophobia Scale. The average score was 102.14 (SD = 24.73), with a median of 104.00. Multivariate logistic regression revealed that lacking a homosexual acquaintance, endorsing environmental (rather than biological) explanations for homosexuality and expressing discomfort working with LGB individuals were associated with higher odds of scoring above the median, as was holding a bachelor’s degree (vs. high school/associate). The final model achieved an overall classification accuracy of 80.0%, correctly identifying 72.0% of those below and 88.0% of those above the median.

The mean homophobia score observed in our study (M = 102.14, SD = 24.73) was higher than those reported in several previous studies conducted in Türkiye using comparable instruments, including Gonenc and Senturk Erenel [44], Çakır and Harmancı Seren [45], and Aydını and Uzun Şahin [46]. Although our mean was lower than that of Soner and Altay [47], the overall pattern reflects a relatively elevated level of homophobia among practicing nurses. This finding is somewhat unexpected considering the study setting—a tertiary university hospital hosting a multidisciplinary gender clinic—where some degree of exposure to gender-diverse individuals might be presumed. While Allport’s contact hypothesis posits that interpersonal interaction reduces prejudice [48], this effect may be nullified when the contact is superficial or unstructured [49]. Our findings suggest that such exposure, when passive or incidental, may be insufficient to mitigate negative beliefs about homosexuality.

Previous evidence suggests that educational attainment is inversely related to homophobia [47,50]. Contrary to this pattern, our regression analysis identified possession of a bachelor’s degree as independently associated with higher rather than lower HRHS scores (OR = 4.24, p = .036). This association was not significant at the bivariate level (p = .291), the overall effect of education did not reach significance in the adjusted model (p = .075), and the contrast emerged only after adjustment; it should therefore be interpreted with caution and may reflect the composition of this sample rather than a genuine effect of formal education. While some prior studies found education-related group differences without retaining this variable in multivariate models [45], education was retained in the model only through the bachelor’s-level contrast, and in the opposite direction. In addition, maternal education was reported as a relevant inverse correlate in previous student-based samples [44], raising the possibility that intergenerational educational influences may also shape attitudes. Taken together, these findings suggest that formal educational attainment alone does not ensure lower prejudice, and point instead to the content of nursing curricula as the relevant target.

Personal acquaintance with a homosexual was significantly associated with lower homophobia in our regression model (OR = 0.19, p = .032), consistent with a growing body of literature that supports the relevance of interpersonal contact in shaping attitudes toward sexual minorities. This finding is consistent with prior studies conducted in Türkiye, including Soner and Altay [47] and Aydını and Uzun Şahin [46], both of which identified direct personal contact—such as having a gay friend—as a meaningful correlate of lower prejudice. Similarly, Gonenc and Senturk Erenel [44] reported substantially lower HRHS scores among nursing students who had a homosexual acquaintance. This relationship, also reported by Çakır and Harmancı Seren [45], was retained in our multivariate model, indicating that the association persists after adjustment for other factors. These converging results lend qualified support to Allport’s contact hypothesis [48], which posits that direct interpersonal contact with members of stigmatized groups fosters empathy and reduces prejudice. This may reflect the more homogenous professional network of our nursing sample and underscores the limited visibility of LGB individuals within clinical environments. Taken together, these findings suggest that even limited interpersonal exposure—when genuine and direct— may function as a buffer against homophobia. As such, creating structured opportunities for healthcare providers to engage meaningfully with LGB individuals may represent a valuable component of anti-stigma interventions in clinical training.

Beliefs regarding the etiology of homosexuality emerged as a strong predictor in our study, with participants endorsing a biological explanation demonstrating significantly lower levels of homophobia (OR = 0.22, p = .013). This finding reinforces earlier observations by Soner and Altay [47], who noted increased prejudice among individuals perceiving homosexuality as either a sin or an illness. Unlike their descriptive-only analysis, our regression-based approach demonstrates that etiological beliefs hold independent predictive power when modeled alongside other variables. This aligns with existing literature suggesting that essentialist or biomedical explanations may reduce moral judgment and stigma by framing homosexuality as an innate, non-volitional characteristic [49].

In our study, higher self-reported comfort working with LGB colleagues was associated with significantly lower odds of being in the higher HRHS score group (OR = 0.41, p = .019). This finding suggests that comfort with LGB coworkers may reflect more than surface-level tolerance; it may indicate the internalization of inclusive values that align with professional ethics and workplace norms. While previous research has examined general willingness to communicate with LGB individuals [47], our findings emphasize a more embedded form of acceptance situated within professional collaboration. Such comfort likely contributes to organizational cultures that are less permissive of stigmatizing beliefs and more supportive of equity. These results are consistent with conceptual models positing that prejudice is shaped not only by individual attitudes but also by the broader institutional and structural environment. Organizational climate theory [51] and structural stigma frameworks [14] both highlight the role of workplace norms, leadership, and policy structures in reinforcing or mitigating bias within clinical settings. Promoting inclusive workplace climates may therefore serve as a structurally grounded strategy to reduce homophobia in healthcare practice.

Despite some degree of exposure to LGB individuals, these findings raise questions about the nature and depth of such contact as well as broader sociocultural influences. Homophobia scores among nurses in Türkiye must be interpreted within a broader sociocultural and institutional context. While interpersonal contact, etiological beliefs, and workplace comfort emerged as significant predictors, some variables—such as marital status—showed notable group-level differences despite lacking statistical significance in the final model. Married nurses, for instance, were more likely to be in the higher HRHS score group at the bivariate level, potentially reflecting the influence of traditional family norms and heteronormative expectations that remain salient in Turkish cultural settings. A similar pattern was seen for parental attitudes, with negative parental views toward LGB individuals more common in the higher HRHS score group at the bivariate level (74.0% vs. 52.0%; p = .038), consistent with the role of familial socialization in shaping such attitudes. Since the 2009 Religious Affairs Council’s declarations and the increasing legal and social restrictions on public expressions of LGB identity—such as the repeated banning of Pride parades—state-level discourse may have contributed to an ambient climate of homophobia that shapes both institutional norms and professional attitudes. This interpretation aligns with Hubbard’s conceptualization of how heteronormative values can be subtly embedded in institutional practices and local governance structures [52] and resonates with Hatzenbuehler et al.’s framework of structural stigma as a fundamental cause of population health inequalities [12]. However, the generalizability of these findings may be limited due to the single-center design, the predominance of female participants, and the inclusion of a specialized gender clinic. These sample characteristics may not reflect the broader nursing population in Türkiye, particularly in more conservative or non-specialized settings.

Although certain background variables—such as marital status, gender, and religious affiliation—did not emerge as statistically significant predictors in our final model, their theoretical relevance warrants attention. The limited variability in these characteristics—especially the predominance of female and Muslim participants—may have restricted the statistical power to detect meaningful effects. Furthermore, their impact may be mediated by more proximal constructs such as education level or institutional climate, which were already captured in the multivariate analysis. Future research with more demographically diverse and representative samples may help uncover these latent associations more clearly.

The presence of a self-identified homosexual nurse with a high homophobia score is particularly striking, suggesting the possibility of internalized stigma—a recognised but frequently overlooked phenomenon in healthcare settings [49]. This underscores the limitations of conventional awareness-based interventions and highlights the necessity of targeting the emotional, relational, and structural layers of prejudice. Professional development initiatives must therefore extend beyond surface-level training, incorporating sustained ethical reflection, culturally competent pedagogy, and longitudinal follow-up to foster meaningful attitudinal change and promote inclusivity in clinical environments.

While the need for structured, evidence-based training to combat homophobia among nurses is evident, our findings also point to deeper systemic challenges in the educational pipeline. The distribution of self-perceived knowledge in our sample is consistent with this: no nurse rated their knowledge of homosexual health as good or expert, and 70% rated it as none or minimal. A national survey by Aslan et al. [53] found that only 23% of nurse educators in Türkiye addressed LGB-related content in their curricula, with over half citing lack of knowledge as the primary barrier.

### Implications for practice and policy

Although educational attainment and personal acquaintance are not themselves readily modifiable, the pattern of associations observed in this study points to potentially modifiable educational and institutional targets. Nursing curricula and continuing professional development may benefit from integrating evidence-based content on sexual orientation, including explicit attention to misconceptions that frame homosexuality as pathological, temporary, or simply a matter of individual choice. The association between personal acquaintance with LGB individuals and lower HRHS scores also suggests a potential role for structured opportunities for meaningful contact within educational settings. At the institutional level, the association between greater comfort working with LGB colleagues and lower HRHS scores highlights workplace climate as another relevant target, including inclusive professional practices and clear non-discrimination standards. Faculty development may be particularly important in Türkiye, given previous evidence that LGB-related content remains limited in nursing education and that nurse educators themselves report knowledge gaps. These implications should be interpreted as potential targets rather than established interventions, given the cross-sectional design of the present study.

The present survey was conducted to inform a subsequent in-service training programme, with all data collected before participants were exposed to the training content; the findings therefore represent a baseline assessment rather than an evaluation of the programme. Nevertheless, the knowledge gaps observed here, together with the associations involving etiological beliefs and workplace comfort, indicate domains that may warrant attention when designing or refining educational initiatives for nurses.

### Strengths

This study possesses several methodological and contextual strengths that enhance the robustness and applicability of its findings. First, the use of multivariate logistic regression allowed for the identification of independent predictors of homophobia, providing a more precise understanding of its underlying determinants than descriptive analyses alone. Second, the study was conducted exclusively with practicing nurses actively engaged in direct patient care, thereby improving ecological validity and ensuring relevance to real-world clinical contexts. Third, the setting—a tertiary university hospital that hosts a multidisciplinary gender clinic—offered a unique institutional environment where incidental exposure to sexual and gender minority patients was likely, potentially shaping professional attitudes. Importantly, the study achieved a 100% response rate through anonymous, self-administered questionnaires completed in private settings, which minimized social desirability bias and enhanced the reliability of responses. Finally, by incorporating both cognitive (e.g., beliefs about the etiology of homosexuality) and affective (e.g., comfort working with LGB colleagues) components, the study provides a multidimensional framework for understanding the drivers of homophobia in healthcare environments. These features collectively support the study’s potential to inform the development of targeted educational interventions within nursing curricula and clinical training programs. Rather than introducing entirely novel individual correlates, the present study contributes by examining several established factors jointly in a multivariable model among practicing clinical nurses in Türkiye, where empirical evidence on homophobia in nursing remains limited.

### Limitations

This study has several limitations that warrant consideration. First, its cross-sectional design precludes causal inference regarding the observed associations. Second, while the data were collected anonymously, the use of self-report measures may have introduced social desirability bias. The 100% response rate—although suggestive of strong institutional engagement and participant cooperation—may also reflect self-selection or social desirability bias due to the sensitive nature of the topic and the at-home completion format. Third, the sample was drawn from a single tertiary university hospital with a multidisciplinary gender clinic, which may limit the generalizability of the findings to other institutional or regional contexts. The modest sample size (N = 100) should also be interpreted in light of the sociocultural context in which the study was conducted, where research on sexual prejudice among healthcare professionals may be shaped by contextual sensitivities. The predominance of female participants (93%) further restricts generalizability and prevented meaningful subgroup analyses by gender. Importantly, this study focused specifically on homophobia, as measured by the Hudson and Ricketts Homophobia Scale, and did not address prejudice against transgender individuals. However, a separate analysis using dedicated transphobia measures from the same sample has been published previously [41]. Dichotomising the homophobia score at the median inevitably discards information, and the resulting threshold is sample-specific rather than normative; analyses using the continuous score in larger samples would allow finer-grained estimation of effect magnitudes. Finally, while the HRHS is a validated measure that captures internalized negative attitudes toward homosexuality, it does not directly assess clinical behaviors or discriminatory practices. Therefore, caution is warranted when extrapolating these findings to patient care. Nevertheless, existing literature suggests that personal prejudice—particularly when unexamined—can subtly influence professional interactions, especially in emotionally or morally charged contexts. Future research should consider mixed-method approaches to explore how internal attitudes translate into actual clinical behavior.

## Conclusions

This study is among the few in Türkiye to empirically examine predictors of homophobia among practicing nurses in a tertiary care hospital setting. Despite being situated within an institution offering gender-focused services, HRHS scores were not correspondingly low, indicating that incidental exposure alone may be insufficient to challenge ingrained societal biases. The independent predictive value of educational attainment, interpersonal contact with homosexual individuals, biological beliefs about etiology, and comfort working with LGB colleagues highlights several factors that merit attention in training and practice. Educational attainment is not itself a modifiable target, but etiological beliefs and workplace comfort indicate domains that nursing education and institutional practice may address. Importantly, these findings do not suggest individual blame, but rather underscore systemic gaps in nursing education and institutional culture that may unintentionally perpetuate stigmatizing beliefs. In this context, addressing homophobia is not only a professional duty but an ethical obligation aligned with the principles of patient dignity, nondiscrimination, and equitable care.

This research contributes to the growing literature by integrating cognitive, affective, and experiential predictors into a multivariate model, offering a foundation for future studies and interventions. While further longitudinal and multicenter research is needed to confirm generalizability, our findings support the inclusion of sexual and gender minority health in nursing curricula and professional development. Aligning education with human rights-based care principles is essential to building a healthcare environment where all patients are treated with respect, regardless of sexual orientation.
